# Population structure and cryptic genetic variation in the mango fruit fly, *Ceratitis cosyra* (Diptera, Tephritidae)

**DOI:** 10.3897/zookeys.540.9618

**Published:** 2015-11-26

**Authors:** Massimiliano Virgilio, Hélène Delatte, Yasinta Beda Nzogela, Christophe Simiand, Serge Quilici, Marc De Meyer, Maulid Mwatawala

**Affiliations:** 1 Royal Museum for Central Africa, Leuvensesteenweg 13, B3080 Tervuren, Belgium; 2 Centre de Coopération Internationale en Recherche Agronomique pour le Développement – Unité Mixte de Recherche Peuplements Végétaux et Bioaggresseurs en Milieu Tropical (CIRAD, UMR PVBMT), Saint-Pierre, La Réunion, France; 3 Department of Crop Science and Production, Sokoine University of Agriculture, Tanzania

**Keywords:** *Ceratitis cosyra*, fruit flies, cryptic species, microsatellites, morphology, sympatric speciation

## Abstract

The fruit fly *Ceratitis cosyra* is an important agricultural pest negatively affecting the mango crop production throughout Africa and also feeding on a variety of other wild and cultivated hosts. The occurrence of deeply divergent haplotypes, as well as extensive morphological variability, previously suggested possible cryptic speciation within *Ceratitis cosyra*. Here we provide the first large-scale characterisation of the population structure of *Ceratitis cosyra* with the main objective of verifying cryptic genetic variation. A total of 348 specimens from 13 populations were genotyped at 16 polymorphic microsatellite loci. Hardy-Weinberg equilibrium (HWE) deviations were observed in 40.4% of locus-population combinations and suggested the occurrence of genetic substructuring within populations. Discriminant Analysis of Principal Components (DAPC) showed genetic divergence between the vast majority of vouchers from Burundi and Tanzania (plus a few outliers from other African countries) and all other specimens sampled. Individual Bayesian assignments confirmed the existence of two main genotypic groups also occurring in sympatry. These data provided further support to the hypothesis that *Ceratitis cosyra* might include cryptic species. However, additional integrative taxonomy, possibly combining morphological, ecological and physiological approaches, is required to provide the necessary experimental support to this model.

## Introduction

The tephritid fly, *Ceratitis cosyra* (Walker, 1849), is possibly the most important indigenous pest of mango throughout sub-Saharan Africa. It is estimated that *Ceratitis cosyra* can reduce the mango crop yield between 20 and 30%, and the damage this pest causes affects the quality and market value of the fruit at both local and international markets (Lux et al. 2003). Although it has been partially displaced by the invasive *Bactrocera dorsalis* (Hendel, 1912) in recent years (Ekesi et al. 2009), it still has serious impact on the mango production, especially for early and mid-season cultivars in western Africa (Vayssières et al. 2009). The range of host records for *Ceratitis cosyra* is relatively large (but not as large as for other congeneric fruit flies such as *Ceratitis rosa* Karsch, 1887 or *Ceratitis capitata* (Wiedemann, 1824), see Copeland et al. 2006 for details) and includes guava as well as a variety of hosts from Anacardiaceae, Annonaceae, Apocynaceae and Rubiaceae among others (De Meyer 1998).

In Kenya, the mango fruit fly can be found in both lowlands and highlands at altitudes between 20 and 2100 m, while Geurts et al. (2012) observed predominance at lower altitudes in Tanzania. On the Kenyan coast, *Ceratitis cosyra* uses wild fruits, primarily the marula tree, *Sclerocarya birrea* (A. Rich.) Hochst., as an alternative host when mango is not available (Copeland et al. 2006). Similarly, in Tanzania *Ceratitis cosyra* shifts to soursop (*Annona muricata* Linnaeus) out of the mango season (Mwatawala et al. 2009). The distribution of *Ceratitis cosyra* in southern Africa is limited to the subtropical regions and its occurrence in this region coincides with the known distribution range of *Sclerocarya birrea* (De Meyer 2001). *Ceratitis cosyra* is commonly intercepted in Europe (Li et al. 2009 and references therein) where the establishment of adventive populations raises serious concerns. Based on the observed altitudinal records, Copeland et al. (2006) suggested that *Ceratitis cosyra* may be pre-adapted to survive in the same subtropical and Mediterranean climatic areas as the cosmopolitan *Ceratitis capitata*, thus representing a potential risk of invasion and establishment of this pest to Europe and the US mainland (but see Grout and Stoltz 2007 for a less pessimistic view on its invasion potential). Similarly, Li et al. (2009) listed North, Central and South American and Middle Eastern, Asian and Australian countries as potential suitable areas for the establishment of adventive *Ceratitis cosyra* populations.

Barr et al. (2012) investigated the utility of DNA barcoding for molecular identification of several tephritid pests, including *Ceratitis cosyra*. They suggested that the mango fruit fly might include cryptic species. In fact, in their study, *Ceratitis cosyra* was represented by a larger haplotype group with vouchers from Mali (two sampling locations) and Kenya (two sampling locations) and by two *Ceratitis cosyra* outliers sampled at the coast of southern Kenya (Shimba Hills). Surprisingly, these two individuals, sharing the same COI haplotype, were separated from the main haplotype group by 52 mutational steps. Also on morphological grounds, *Ceratitis cosyra* has been the subject of confusion. Various taxa (now considered synonyms) have been described as separate species or varieties based on differences in cephalic and leg chaetotaxy and mesonotal patterns (see De Meyer 1998 for detailed discussion). The occurrence of cryptic species in *Ceratitis cosyra* would pose relevant issues with respect to pest management, ecological modelling and estimation of its invasion potential. In this study, the population structure of *Ceratitis cosyra* was inferred across the species distributional range as a first step towards exploring its cryptic diversity.

## Methods

A total of 348 specimens of *Ceratitis cosyra* from 13 populations (13 < n < 32) were collected in Africa from 2000 to 2012 (Table 1, see also supplementary file SF 1: Map of sampling locations). DNA was extracted from ethanol preserved adults by the DNeasy Blood and Tissue Kit (Qiagen) as per the manufacturer’s instructions. Individual flies were genotyped at 16 polymorphic microsatellite loci developed by Delatte et al. (2014): Co1350, Co1444, Co2J, Co486, Co633, Co806, CoD4, CoES, CoKW, CoOI, CoP7, CoQT, CoRTA, CoWU, CoZ29, CoZW (see Delatte et al. (2014) for primer sequences and laboratory procedures). Electrophoretic analyses were conducted on an automated ABI Prism 3100 Genetic Analyzer (Applied Biosystem) with individuals declared non-amplifiable at a locus after two independent amplification failures. The genotypes of the 348 individual insects were analysed by the ADEGENET 1.4-2 package of the R statistical software (Jombart 2008) to ascertain the genetic variability and differentiation, among the *Ceratitis cosyra* populations, including number of alleles per locus (N_all_), observed and expected heterozygosity (H_obs_, H_exp_) and deviations from the Hardy-Weinberg equilibrium (HWE). The function *genotype_curve* of the R package POPR (Kamvar et al. 2014) was used to calculate a genotype accumulation curve (this function randomly sample loci without replacement and count for the number of multilocus genotypes). Linkage disequilibrium was tested for each population across each pair of loci using the log likelihood ratio statistic implemented in GENEPOP 4.3 (Rousset 2008) and assessing significance through Markov-chain randomizations based on 1000 dememorizations, 100 batches, and 5000 iterations per batch. FreeNA 1.0 (Chapuis and Estoup 2007) was used to estimate null allele frequencies (per locus and population) according to Dempster et al. (1977). Probability values of repeated tests were corrected for Type I errors using the False Discovery Rate (FDR) procedure (Benjamini and Hochberg 1995). Isolation by distance (IBD) was verified in ADEGENET through Mantel test between Edwards’ genetic distances and Euclidean geographic distances (1000 permutations). Principal Component Analysis (PCA) was used to ordinate specimens in multivariate space. Prior to PCA, the SCALEGEN function of ADEGENET was used to centre the data and replace missing genotypes with mean allele frequencies. Specimens from different populations were then ordinated by maximising between-group variances through Discriminant Analysis of Principal Components (DAPC). The number of Principal Components (PCs) retained in DAPC was optimised using XVALDAPC function of ADEGENET (Jombart et al. 2010).

**Table 1.** Population locations and genetic variability. Sampling locations, geographic coordinates (decimal degrees) and summary of genetic variability in 13 populations of *Ceratitis cosyra* (see Figure 1) genotyped at 16 microsatellite loci. N: number of individuals per population, N_all_: total number of alleles, H_obs_: observed heterozygosity, H_exp_: expected heterozygosity, null: mean null allele frequency based on Dempster et al. (1977). Standard deviations in parentheses.

	Locality		Latitude	Longitude	N	N_all_	H_obs_	H_exp_	null
1	Burkina Faso	(interception)			29	67	0.412 (0.319)	0.484 (0.289)	0.060 (0.094)
2	Burundi	Isabu	-3.394	29.361	32	79	0.391 (0.312)	0.478 (0.304)	0.070 (0.090)
3	Ethiopia	Badano	9.317	41.217	13	74	0.430 (0.313)	0.475 (0.279)	0.065 (0.073)
4	Ivory Coast	Korhogo	9.450	-5.633	18	100	0.417 (0.327)	0.449 (0.366)	0.036 (0.059)
5	Kenya	Nairobi	-1.283	36.817	32	122	0.415 (0.276)	0.557 (0.266)	0.109 (0.062)
6	Malawi	Zomba	-15.383	35.333	29	118	0.481 (0.266)	0.601 (0.266)	0.082 (0.090)
7	Mali	(interception)			29	69	0.372 (0.308)	0.432 (0.331)	0.050 (0.073)
8	Mozambique	Cuamba	-14.816	36.535	32	124	0.478 (0.276)	0.581 (0.255)	0.076 (0.092)
9	Nigeria	Sokoto	13.051	5.231	26	93	0.421 (0.292)	0.482 (0.283)	0.059 (0.074)
10	South Africa	Constantia	-23.644	30.679	22	110	0.504 (0.284)	0.570 (0.278)	0.061 (0.075)
11	Senegal	Sané	12.750	-15.500	28	114	0.393 (0.262)	0.559 (0.259)	0.112 (0.112)
12	Sudan	Singa	13.150	33.850	32	113	0.376 (0.287)	0.532 (0.279)	0.123 (0.091)
13	Tanzania	Mzinga	-6.883	37.617	26	101	0.410 (0.255)	0.637 (0.186)	0.146 (0.113)

STRUCTURE 2.3.4 (Pritchard et al. 2000) was used to calculate individual admixture coefficients (Q) across individuals and populations. STRUCTURE analyses were based on the admixture model (individuals were allowed to have mixed ancestries from different clusters) with correlated allele frequencies (allele frequencies in different clusters were likely to be similar due to migration or shared ancestry) and the parameter of the Dirichlet distribution of allelic frequencies (λ) separately inferred for each population. We used STRUCTURE HARVESTER 0.6.94 (Earl and vonHoldt 2012) to infer the optimal number of clusters (K) using the Evanno et al. (2005) parameters. Since this method only detects the uppermost level of population structure when different hierarchical levels exist, we further investigated the genetic substructuring of our dataset by following the sequential clustering method described in Coulon et al. (2008). For this purpose, replicated STRUCTURE runs were performed by (a) dividing the main dataset in subsets of data including individuals assigned to the same cluster, (b) recalculating the optimal K value (Evanno et al. 2005) of each subset of data and (c) repeating the STRUCTURE analyses of each subset of data. We set Q = 0.7 as an arbitrary threshold for cluster assignment and individuals not reaching the threshold were discarded from further replicated runs. For each value of K, five iterations were run for 3 million generations (with 1.5 million generations as burn-in) and the posterior estimates of cluster memberships of the 3 runs with the highest estimated log probability of the data were summarized in CLUMPP 1.1.2 (Jakobsson and Rosenberg 2007) and visualized in DISTRUCT 1.1 (Rosenberg 2004).

## Results

The amount of scored multilocus genotypes reached a plateau after 5-7 sampled loci, indicating that the genetic variability of *Ceratitis cosyra* was adequately sampled by the 16 microsatellites markers used (see supplementary file SF 2: Genotype accumulation curve). The total scored number of alleles (N_all_) ranged from 67 (in the Burkina Faso population) to 124 (Mozambique), with an average proportion of missing data per population ranging from 2.2% (SE = 1.5%, Mali) to 30.9% (SE = 9.0%, Ivory Coast). H_obs _ranged from 0.372 (Mali) to 0.504 (South Africa), while H_exp _from 0.432 (Mali) to 0.637 (Tanzania) (Table 1). Pearson’s Chi-squared test showed significant HWE deviations in 84 of 208 locus-population combinations, corresponding to 40.4% of observations (see supplementary files SF 3: Pearson’s Chi-squared test for Hardy-Weinberg equilibrium and SF 4: Observed and expected heterozygosity). These HWE deviations are compatible with the occurrence of genetic substructuring within populations (Walhund effect) as described below. The average estimated proportion of null alleles was 8.1% (SE = 0.6%) (see supplementary file SF 5: Estimated null allele proportions) and linkage disequilibrium was observed in 16.7% of pairwise tests (see supplementary file SF 6: Linkage disequilibrium). Mantel test did not evidence significant correlation between individual geographic and genetic distances (p > 0.05, see supplementary file SF 7: Mantel test).

**Figure 1.**
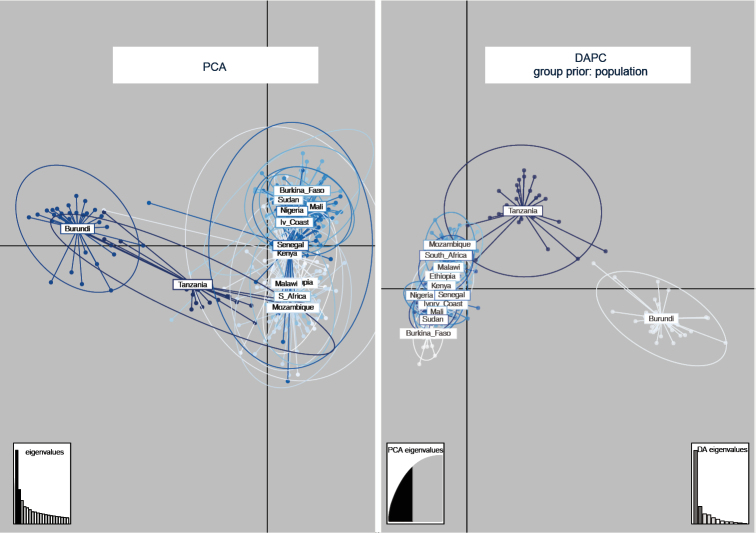
Unconstrained and constrained ordination. Principal Component Analysis (PCA) and Discriminant Analysis of Principal Components (DAPC) of 348 *Ceratitis cosyra* microsatellite genotypes. Specimen groups are labelled inside their 95% inertia ellipses and genotypes are connected to the corresponding group centroids.

PCA was based on 28 PC axes that accounted for 70.6% of cumulative inertia. The first two PCs (Figure 1) represented a relatively low amount of variation (22.3%) and didn’t allow proper resolution of populations (see 95% confidence ellipses). DAPC considered the populations as a priori defined groups and was based on 90 PCs. Stressing the ordination of points by using a prior group only allowed a better resolution of the population from Burundi and, possibly, of that from Tanzania. The latter was clearly separated from the other populations only when excluding 4 STRUCTURE outliers from DAPC (see below). Confidence ellipses of all other populations were largely overlapping (Figure 1).

**Figure 2.**
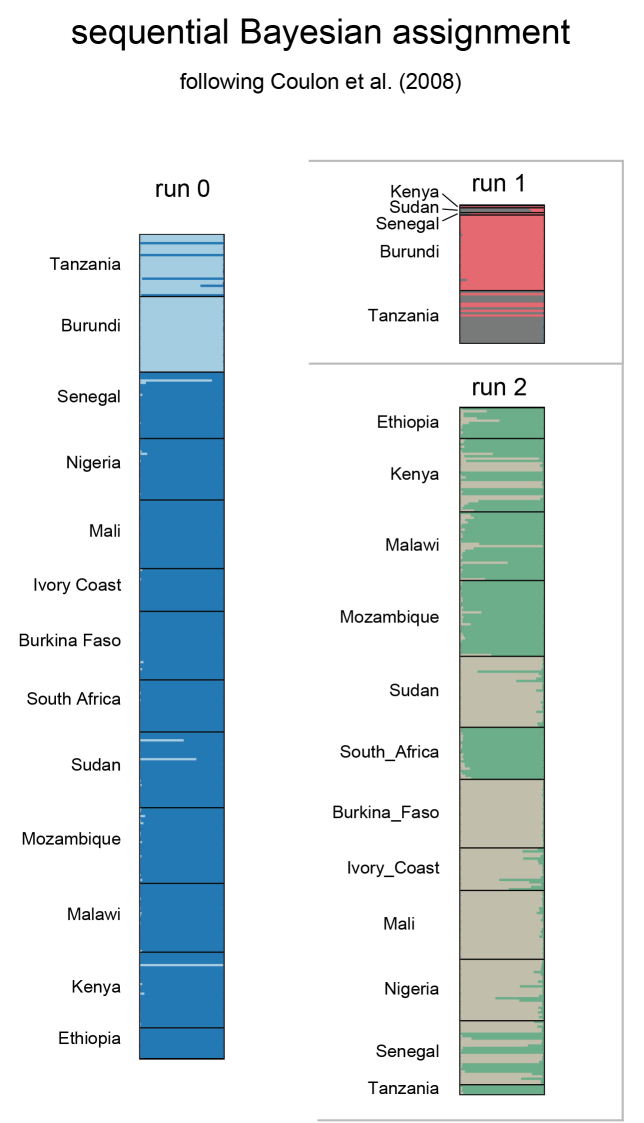
Individual Bayesian assignments. STRUCTURE sequential individual assignments of 348 specimens of *Ceratitis cosyra* from 13 African countries.

The STRUCTURE analysis of the entire dataset (n=345, run 0, Figure 2) showed ∆K values (Evanno et al. 2005) peaking at K=2 indicating that the main hierarchical level of the population structure is based on 2 genotype groups. A first and smaller group (then separately analysed in run 1) included all specimens from Burundi (n=32) and 22 out of the 26 individuals from Tanzania. A second and larger group (subsequently analysed in run 2) included 98.6% of genotypes from all other populations combined (n=290), including the 4 outliers from Tanzania (see supplementary file SF 8: STRUCTURE sequential assignments). Run 1 (K=2) resolved all specimens from Burundi in one group (that also included one outlier from Kenya and one from Senegal), while specimens from Tanzania were partially assigned to the Burundi group (5 specimens) and, for a larger part, to a second group (17 specimens) together with 2 outliers from Sudan. Run 2 (K=2) resolved specimens from South Africa (100%), Mozambique (96.9%), Malawi (93.1) and Ethiopia (84.6%) in a first group and specimens from Burkina Faso (100%), Mali (100%), Nigeria (96.2%), Sudan (87.5%) and Ivory Coast (88.9%) in a second group. Populations from Senegal and Kenya included specimens that were assigned in part to the first group (35.7% and 62.5%, respectively) and in part to the second (60.7% and 31.3%).

## Discussion

The morphospecies *Ceratitis cosyra* includes two groups of genetically well-differentiated individuals. The vast majority of vouchers from Burundi and Tanzania (plus a few outliers from other African countries) belong to the first of these two groups, all other specimens to the second. Specimens from the two groups were also found sympatrically in a number of populations from Kenya, Senegal, Sudan, and Tanzania. Interestingly, the two *Ceratitis cosyra* outliers described by Barr et al. (2012) were also sampled from the Kenyan coast. The fact that our Kenyan population included specimens from the two clusters suggests that Barr et al. might have genotyped representatives of both types. If this would be confirmed, the two genotypes resolved trough microsatellite markers might also show marked differentiation in the cytochrome oxidase I gene barcode region so that they might be more easily diagnosed through DNA barcoding rather than through microsatellite genotyping.

Further studies are needed to verify if specimens from the two genotypic clusters are also morphologically, ecologically and / or physiologically different and to which extent the two groups are connected by gene flow. A preliminary screening of thorax patterns of the samples used in this study (6 characters scored, data not shown) did not reveal straightforward morphological differences between groups. Wing morphometrics (Van Cann et al. 2015) might provide a more suitable tool to further investigate morphological cryptic variation in *Ceratitis cosyra*.

A wide variety of hosts have been described for *Ceratitis cosyra*, including Annonaceae (such as the introduced soursop) or Anacardiaceae (including the indigenous marula or the introduced mango). An intriguing hypothesis is that the two different *Ceratitis cosyra* types might also have different host preferences, similarly to what has been observed by McPheron et al. (1988) for *Rhagoletis pomonella* (Walsh, 1867). A separate host range characterization for the two types might provide useful information and help to understand if the observed genetic split has a recent evolutionary history (possibly related to the introduction of novel hosts in Africa) or a deeper evolutionary origin.

The sequential Bayesian assignment of genotypes also helped to disentangle the effects of cryptic speciation and of population structure within each of the genotypic groups. Specimens from Burundi and Tanzania are, to a less extent, genetically divergent, and among samples from the other African countries, two groups can be further resolved. In the latter case, specimens could be roughly subdivided between Western African samples (including Burkina Faso, Ivory Coast, Mali and Nigeria) and Eastern / Southern African samples (including Ethiopia, Tanzania, Malawi, Mozambique, South Africa) with the notable exception of Sudan (which is genetically closer to the West African samples) and of Kenya and Senegal (that included a mix of individuals from both groups). Morphological differences were considered by De Meyer (1998) to represent a plausible (but incomplete) geographical split between western and eastern Africa, and this is also only partially corroborated by the genotypic clustering of this study. On the other hand the preliminary morphological screening of thoracic patterns does not provide any support for this division. Most importantly, it is not clear to what extent the mixed patterns of Kenya and Senegal and Tanzania can be related to historical evolutionary processes or to more recent events involving fruit trade and transport (Malacrida et al. 2007).

## Conclusions

Marked and sympatric genetic splits are compatible with the occurrence of presumptive cryptic species, within *Ceratitis cosyra*. Additional integrative taxonomy, possibly combining morphological, ecological and physiological data (e.g., see Schutze et al. 2015) is now required to further support this model.

## Supplementary materials

Supplementary material 1 Map of sampling locations Data type: species data This dataset is made available under the Open Database License (http://opendatacommons.org/licenses/odbl/1.0/). The Open Database License (ODbL) is a license agreement intended to allow users to freely share, modify, and use this Dataset while maintaining this same freedom for others, provided that the original source and author(s) are credited.Massimiliano Virgilio, Hélène Delatte, Yasinta Beda Nzogela, Christophe Simiand, Serge Quilici, Marc De Meyer, Maulid Mwatawala

Supplementary material 2 Genotype accumulation curve Data type: data analysis This dataset is made available under the Open Database License (http://opendatacommons.org/licenses/odbl/1.0/). The Open Database License (ODbL) is a license agreement intended to allow users to freely share, modify, and use this Dataset while maintaining this same freedom for others, provided that the original source and author(s) are credited.Massimiliano Virgilio, Hélène Delatte, Yasinta Beda Nzogela, Christophe Simiand, Serge Quilici, Marc De Meyer, Maulid Mwatawala

Supplementary material 3 Pearson’s Chi-squared test for Hardy-Weinberg equilibrium Data type: data analysis This dataset is made available under the Open Database License (http://opendatacommons.org/licenses/odbl/1.0/). The Open Database License (ODbL) is a license agreement intended to allow users to freely share, modify, and use this Dataset while maintaining this same freedom for others, provided that the original source and author(s) are credited.Massimiliano Virgilio, Hélène Delatte, Yasinta Beda Nzogela, Christophe Simiand, Serge Quilici, Marc De Meyer, Maulid Mwatawala

Supplementary material 4 Observed and expected heterozygosity Data type: species data This dataset is made available under the Open Database License (http://opendatacommons.org/licenses/odbl/1.0/). The Open Database License (ODbL) is a license agreement intended to allow users to freely share, modify, and use this Dataset while maintaining this same freedom for others, provided that the original source and author(s) are credited.Massimiliano Virgilio, Hélène Delatte, Yasinta Beda Nzogela, Christophe Simiand, Serge Quilici, Marc De Meyer, Maulid Mwatawala

Supplementary material 5 Estimated null allele proportions Data type: data analysis This dataset is made available under the Open Database License (http://opendatacommons.org/licenses/odbl/1.0/). The Open Database License (ODbL) is a license agreement intended to allow users to freely share, modify, and use this Dataset while maintaining this same freedom for others, provided that the original source and author(s) are credited.Massimiliano Virgilio, Hélène Delatte, Yasinta Beda Nzogela, Christophe Simiand, Serge Quilici, Marc De Meyer, Maulid Mwatawala

Supplementary material 6 Linkage disequilibrium Data type: data analysis This dataset is made available under the Open Database License (http://opendatacommons.org/licenses/odbl/1.0/). The Open Database License (ODbL) is a license agreement intended to allow users to freely share, modify, and use this Dataset while maintaining this same freedom for others, provided that the original source and author(s) are credited.Massimiliano Virgilio, Hélène Delatte, Yasinta Beda Nzogela, Christophe Simiand, Serge Quilici, Marc De Meyer, Maulid Mwatawala

Supplementary material 7 STRUCTURE sequential assignments Data type: data analysis This dataset is made available under the Open Database License (http://opendatacommons.org/licenses/odbl/1.0/). The Open Database License (ODbL) is a license agreement intended to allow users to freely share, modify, and use this Dataset while maintaining this same freedom for others, provided that the original source and author(s) are credited.Massimiliano Virgilio, Hélène Delatte, Yasinta Beda Nzogela, Christophe Simiand, Serge Quilici, Marc De Meyer, Maulid Mwatawala
