# Supplementary figures and images for: Population structure and cryptic genetic variation in the mango fruit fly, Ceratitis cosyra (Diptera, Tephritidae)
Source: Zookeys. 2015 Nov 26;(540):525–38. doi: 10.3897/zookeys.540.9618 (PMC4714086; doi:10.3897/zookeys.540.9618)

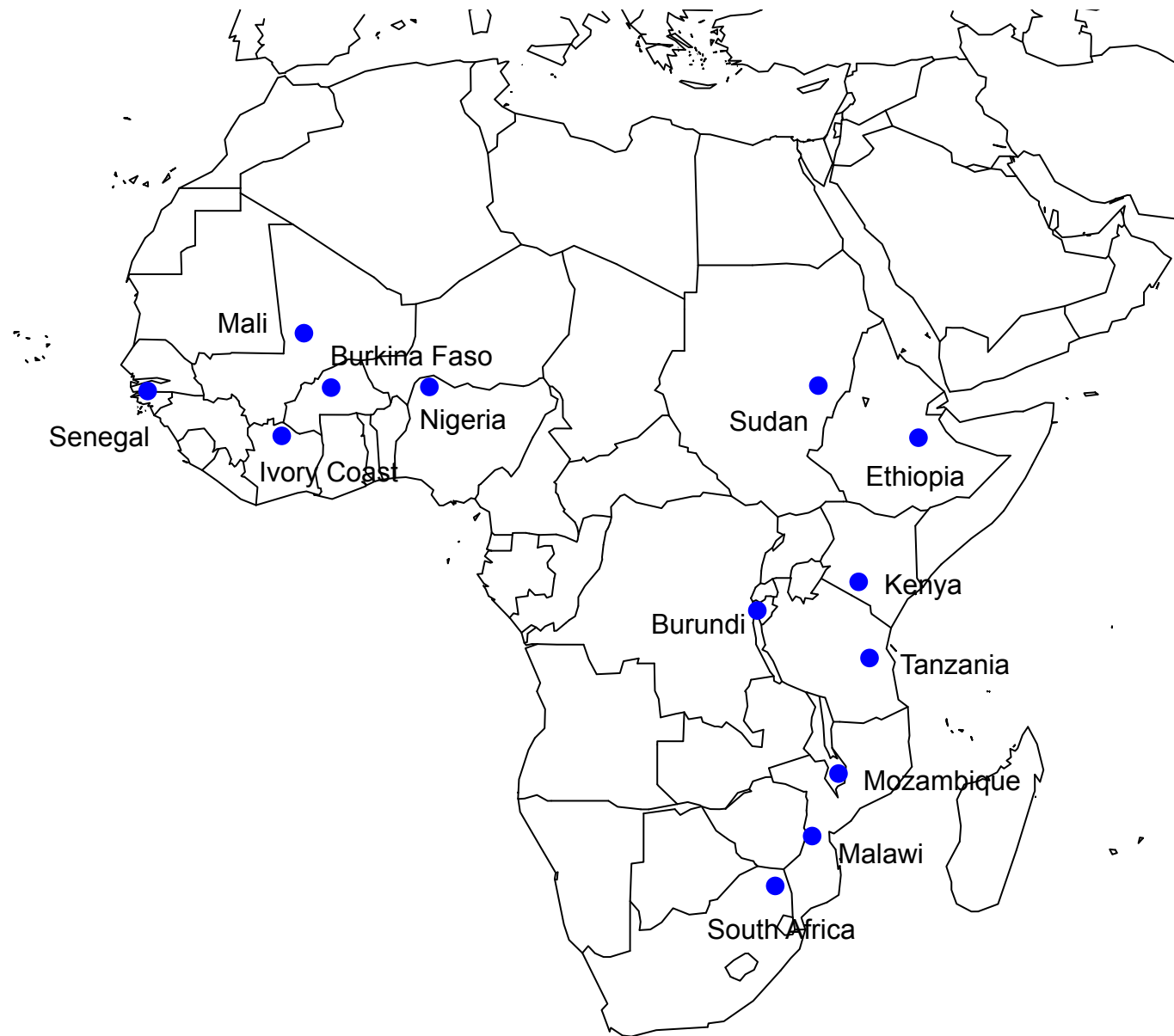

Supplement: Supplementary material 1 — Map of sampling locations [file zookeys-540-525-s001.pdf]

Genotype accumulation curve

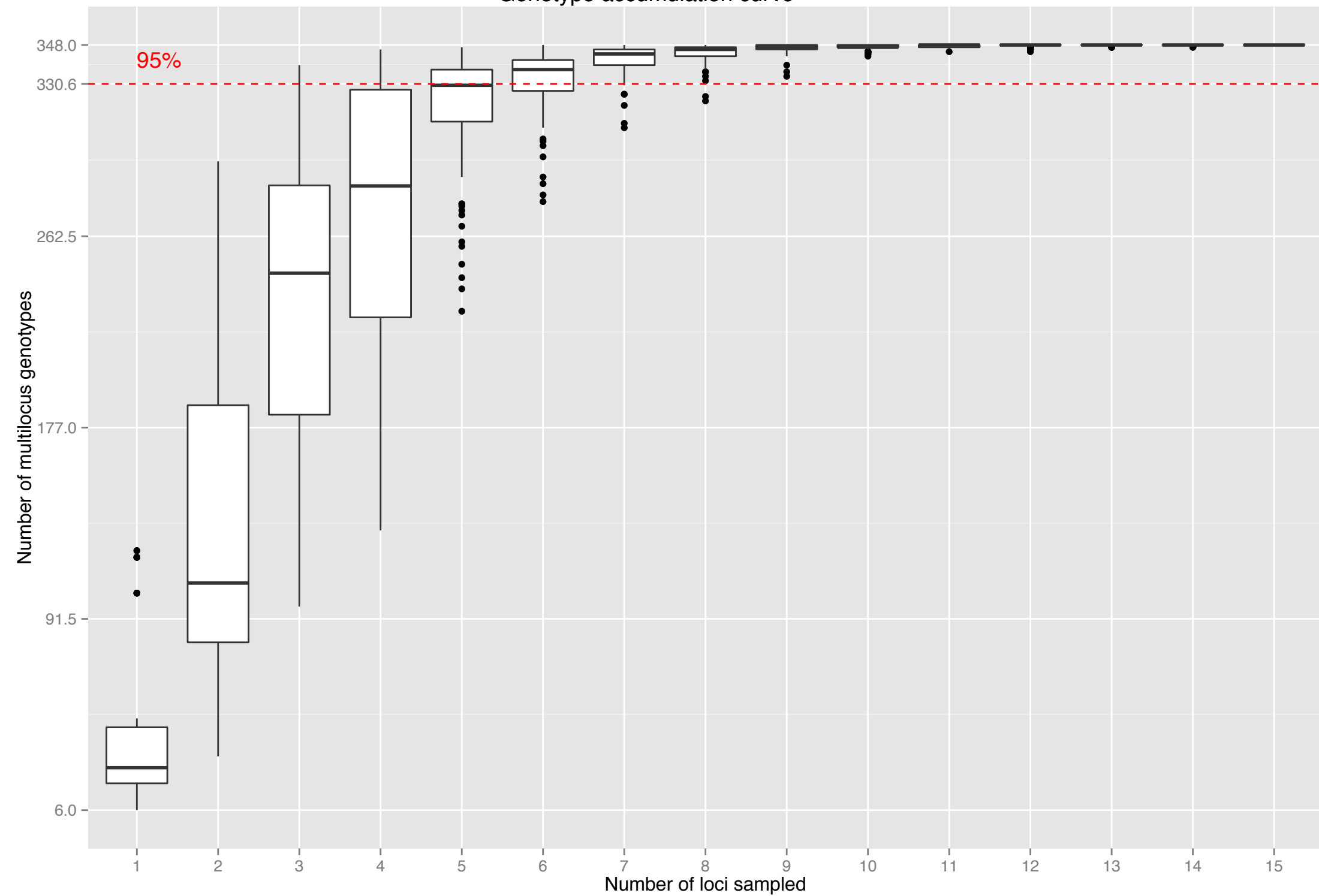

Supplement: Supplementary material 2 — Genotype accumulation curve [file zookeys-540-525-s002.pdf]

# Heterozigosity per locus

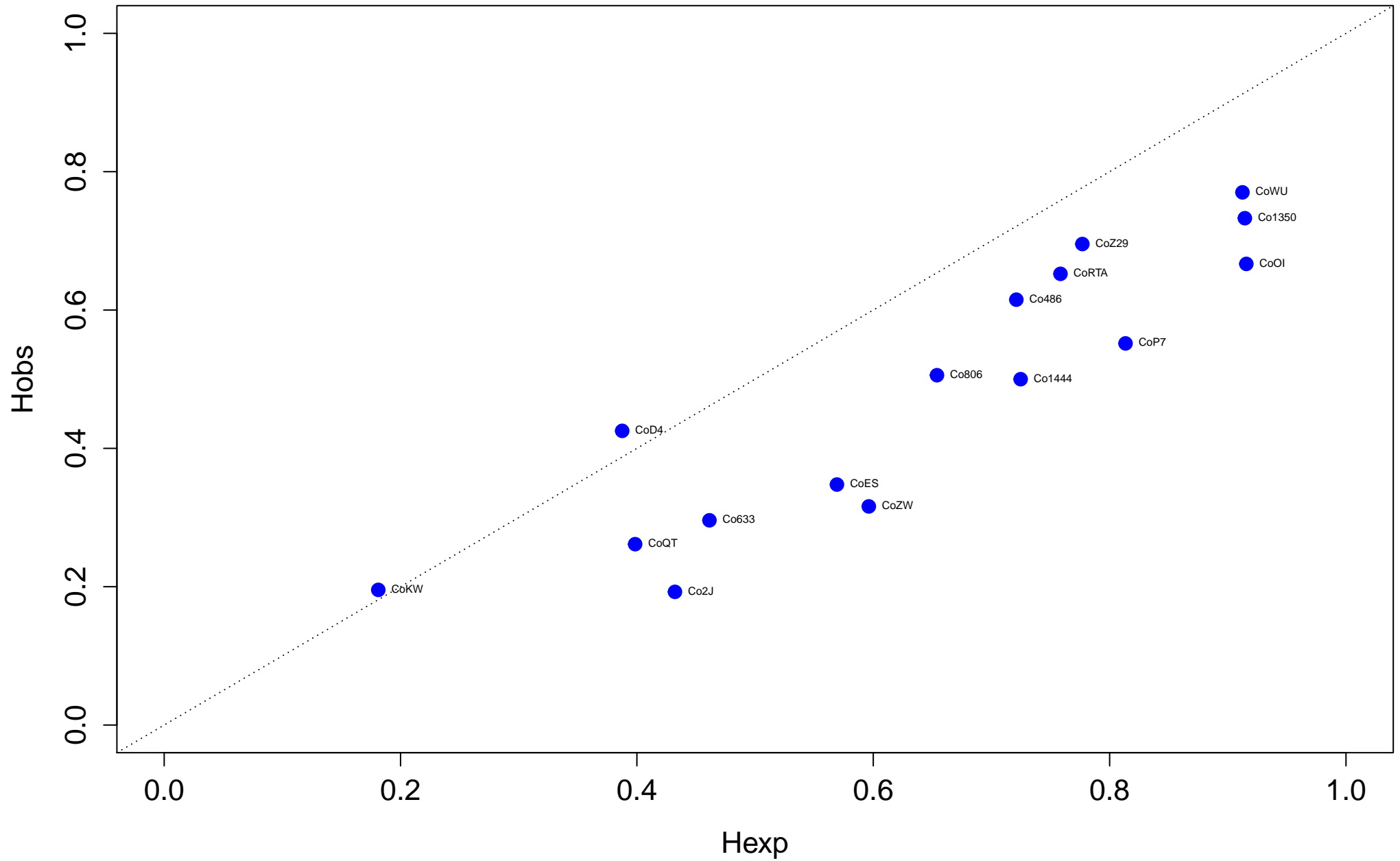

Supplement: Supplementary material 4 — Observed and expected heterozygosity [file zookeys-540-525-s004.pdf]

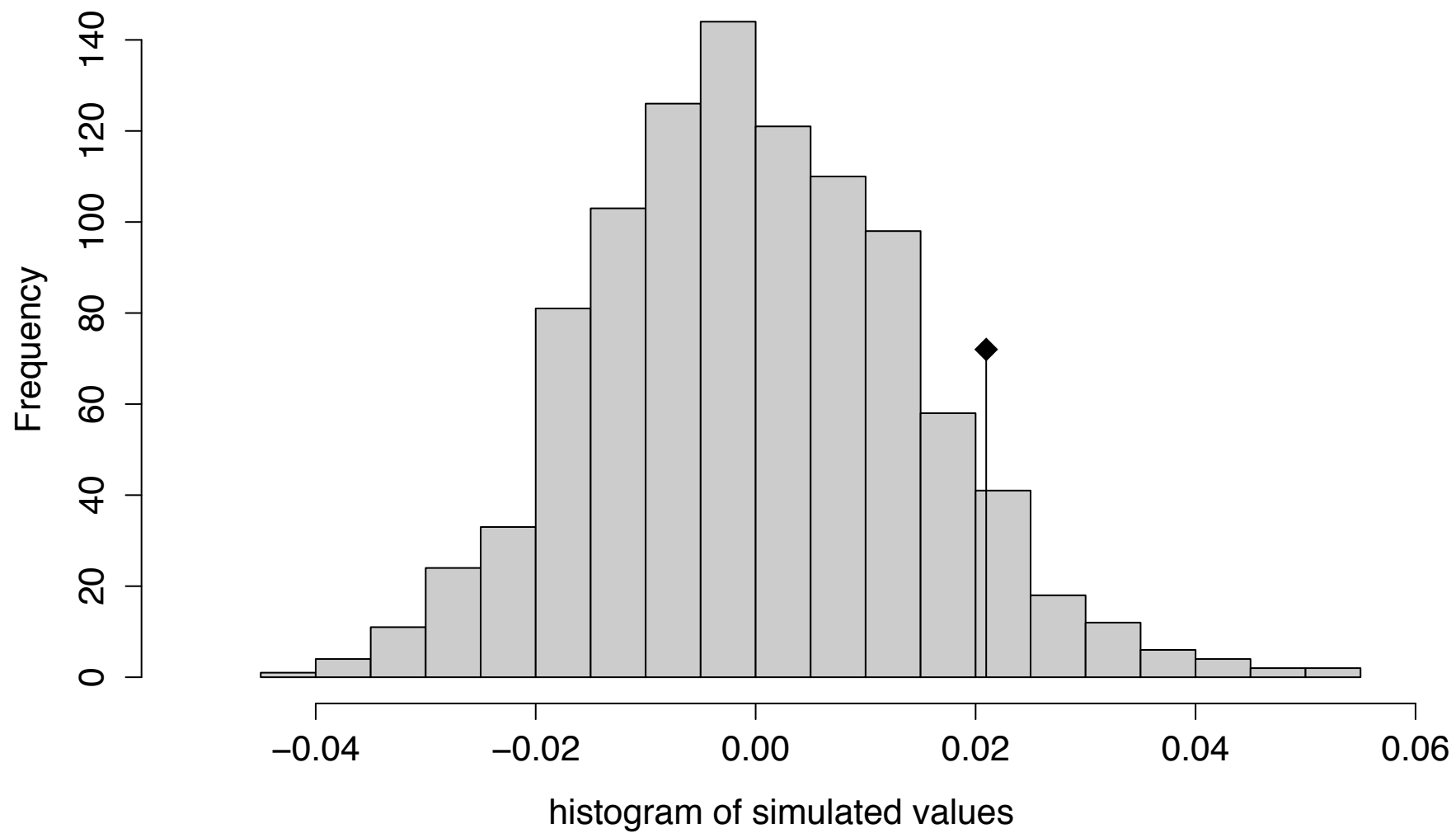

Supplement: Supplementary material 7 — STRUCTURE sequential assignments [file zookeys-540-525-s007.pdf]
