# Supplementary material for: Population structure and cryptic genetic variation in the mango fruit fly, Ceratitis cosyra (Diptera, Tephritidae)
Source: Zookeys. 2015 Nov 26;(540):525–38. doi: 10.3897/zookeys.540.9618 (PMC4714086; doi:10.3897/zookeys.540.9618)
Supplement: Supplementary material 3 — Pearson’s Chi-squared test for Hardy-Weinberg equilibrium [file zookeys-540-525-s003.docx]

SF3: Pearson's Chi-squared test for Hardy-Weinberg equilibrium (per locus, in each population,). Probability values are adjusted following False Discovery Rate correction. *: p<0.05.

|  | Co1350 | Co1444 | Co2J | Co486 | Co633 | Co806 | CoD4 | CoES | CoKW | CoOI | CoP7 | CoQT | CoRTA | CoWU | CoZ29 | CoZW |
| --- | --- | --- | --- | --- | --- | --- | --- | --- | --- | --- | --- | --- | --- | --- | --- | --- |
| Burkina Faso | * |  | * | * |  | * |  |  | * | * | * | * | * | * | * | * |
| Burundi |  |  |  | * |  | * |  |  |  |  | * | * | * |  |  |  |
| Ethiopia | * | * |  |  |  |  |  |  |  |  |  |  |  |  |  |  |
| Ivory Coast | * |  |  |  |  |  |  |  |  |  | * |  |  |  |  |  |
| Kenya | * | * | * |  |  |  |  | * | * |  |  | * | * |  |  |  |
| Malawi |  |  |  |  |  | * |  |  |  | * | * | * |  |  |  |  |
| Mali | * |  |  |  |  | * |  |  |  | * | * |  |  | * |  |  |
| Mozambique |  | * |  |  |  | * | * | * |  |  | * | * |  |  |  | * |
| Nigeria |  | * | * |  |  | * |  |  |  | * |  |  |  |  |  |  |
| South Africa |  |  | * |  |  | * | * |  |  | * |  |  |  |  |  | * |
| Senegal |  | * | * |  | * | * |  |  | * |  | * | * |  |  |  | * |
| Sudan | * | * | * | * | * | * | * |  | * | * | * | * | * |  |  | * |
| Tanzania | * |  | * | * |  |  |  | * |  | * | * | * | * | * |  | * |
| proportion of populations not in HW equilibrium | 0.62 | 0.31 | 0.23 | 0.54 | 0.31 | 0.69 | 0.69 | 0.69 | 0.31 | 0.46 | 0.62 | 0.54 | 0.23 | 0.08 | 0.23 | 0.38 |
