# Supplementary material for: Population structure and cryptic genetic variation in the mango fruit fly, Ceratitis cosyra (Diptera, Tephritidae)
Source: Zookeys. 2015 Nov 26;(540):525–38. doi: 10.3897/zookeys.540.9618 (PMC4714086; doi:10.3897/zookeys.540.9618)
Supplement: Supplementary material 5 — Estimated null allele proportions [file zookeys-540-525-s005.docx]

SF5: Estimated null allele proportions (per locus in each population) according to the expectation maximization (EM) algorithm of FreeNA (Chapuis & Estoup 2007).

|  | CoQT | CoD4 | CoWU | Co2J | CoKW | CoP7 | CoOI | Co806 | Co486 | Co1444 | CoZW | Co1350 | Co633 | CoZ29 | CoES | CoRTA | average | SE |
| --- | --- | --- | --- | --- | --- | --- | --- | --- | --- | --- | --- | --- | --- | --- | --- | --- | --- | --- |
| Burkina Faso | 0.103 | 0.000 | 0.222 | 0.000 | 0.182 | 0.000 | 0.035 | 0.062 | 0.000 | 0.061 | 0.300 | 0.000 | 0.001 | 0.000 | 0.001 | 0.000 | 0.060 | 0.024 |
| Burundi | 0.242 | 0.067 | 0.000 | 0.001 | 0.001 | 0.073 | 0.031 | 0.254 | 0.151 | 0.000 | 0.000 | 0.000 | 0.142 | 0.005 | 0.000 | 0.158 | 0.070 | 0.023 |
| Ethiopia | 0.001 | 0.000 | 0.078 | 0.188 | 0.001 | 0.021 | 0.176 | 0.161 | 0.000 | 0.129 | 0.144 | 0.108 | 0.000 | 0.000 | 0.024 | 0.012 | 0.065 | 0.018 |
| Ivory Coast | 0.000 | 0.000 | 0.000 | 0.000 | 0.001 | 0.115 | 0.193 | 0.001 | 0.023 | - | 0.091 | 0.094 | 0.000 | 0.000 | 0.000 | 0.022 | 0.036 | 0.015 |
| Kenya | 0.147 | 0.103 | 0.070 | 0.135 | 0.178 | 0.116 | 0.040 | 0.049 | 0.000 | 0.233 | 0.155 | 0.122 | 0.088 | 0.105 | 0.182 | 0.026 | 0.109 | 0.016 |
| Malawi | 0.082 | 0.082 | 0.000 | 0.000 | 0.001 | 0.159 | 0.183 | 0.329 | 0.122 | 0.137 | 0.119 | 0.029 | 0.026 | 0.030 | 0.000 | 0.008 | 0.082 | 0.023 |
| Mali | 0.001 | 0.000 | 0.032 | 0.000 | 0.001 | 0.165 | 0.225 | 0.129 | 0.063 | 0.000 | 0.150 | 0.000 | 0.001 | 0.026 | 0.001 | 0.011 | 0.050 | 0.018 |
| Mozambique | 0.133 | 0.000 | 0.012 | 0.015 | 0.001 | 0.139 | 0.082 | 0.270 | 0.049 | 0.231 | 0.208 | 0.004 | 0.000 | 0.000 | 0.065 | 0.000 | 0.076 | 0.023 |
| Nigeria | 0.066 | 0.057 | 0.000 | 0.000 | 0.001 | 0.034 | 0.220 | 0.204 | 0.000 | 0.160 | 0.091 | 0.035 | 0.000 | 0.000 | 0.073 | 0.000 | 0.059 | 0.019 |
| South Africa | 0.000 | 0.000 | 0.045 | 0.146 | 0.001 | 0.000 | 0.131 | 0.244 | 0.048 | 0.086 | 0.181 | 0.031 | 0.019 | 0.038 | 0.000 | 0.000 | 0.061 | 0.019 |
| Senegal | 0.000 | 0.000 | 0.070 | 0.099 | 0.143 | 0.109 | 0.165 | 0.330 | 0.049 | 0.351 | 0.168 | 0.065 | 0.224 | 0.000 | 0.025 | 0.000 | 0.112 | 0.028 |
| Sudan | 0.211 | 0.084 | 0.044 | 0.000 | 0.178 | 0.187 | 0.146 | 0.345 | 0.044 | 0.120 | 0.208 | 0.172 | 0.103 | 0.000 | 0.060 | 0.066 | 0.123 | 0.023 |
| Tanzania | 0.253 | 0.133 | 0.090 | 0.362 | 0.251 | 0.316 | 0.078 | 0.001 | 0.110 | 0.109 | 0.119 | 0.148 | 0.000 | 0.050 | 0.282 | 0.034 | 0.146 | 0.028 |
| average | 0.095 | 0.040 | 0.051 | 0.073 | 0.072 | 0.110 | 0.131 | 0.183 | 0.051 | 0.135 | 0.149 | 0.062 | 0.046 | 0.020 | 0.055 | 0.026 | 0.081 |  |
| SE | 0.026 | 0.013 | 0.016 | 0.030 | 0.026 | 0.024 | 0.019 | 0.033 | 0.013 | 0.027 | 0.019 | 0.016 | 0.019 | 0.008 | 0.023 | 0.012 |  | 0.006 |
