# Supplementary material for: Population structure and cryptic genetic variation in the mango fruit fly, Ceratitis cosyra (Diptera, Tephritidae)
Source: Zookeys. 2015 Nov 26;(540):525–38. doi: 10.3897/zookeys.540.9618 (PMC4714086; doi:10.3897/zookeys.540.9618)
Supplement: Supplementary material 6 — Linkage disequilibrium [file zookeys-540-525-s006.docx]

SF6: Probability tests for linkage disequilibrium among 16 microsatellite loci. *: p<0.05 after False Discovery Rate correction.

|  | CoQT | CoD4 | CoWU | Co2J | CoKW | CoP7 | CoOI | Co806 | Co486 | Co1444 | CoZW | Co1350 | Co633 | CoZ29 | CoES |
| --- | --- | --- | --- | --- | --- | --- | --- | --- | --- | --- | --- | --- | --- | --- | --- |
| CoD4 |  |  |  |  |  |  |  |  |  |  |  |  |  |  |  |
| CoWU |  |  |  |  |  |  |  |  |  |  |  |  |  |  |  |
| Co2J | * |  | * |  |  |  |  |  |  |  |  |  |  |  |  |
| CoKW | * |  |  | * |  |  |  |  |  |  |  |  |  |  |  |
| CoP7 |  |  |  |  |  |  |  |  |  |  |  |  |  |  |  |
| CoOI |  |  |  | * |  | * |  |  |  |  |  |  |  |  |  |
| Co806 |  |  |  |  |  |  |  |  |  |  |  |  |  |  |  |
| Co486 |  |  |  |  |  |  |  | * |  |  |  |  |  |  |  |
| Co1444 |  |  |  | * |  |  |  |  |  |  |  |  |  |  |  |
| CoZW |  |  |  | * |  |  |  |  |  | * |  |  |  |  |  |
| Co1350 |  |  |  |  |  | * |  |  |  |  |  |  |  |  |  |
| Co633 |  |  |  |  | * |  |  |  |  |  |  |  |  |  |  |
| CoZ29 |  |  |  |  |  |  |  |  |  |  |  |  |  |  |  |
| CoES |  |  |  |  |  | * |  |  | * |  |  |  | * |  |  |
| CoRTA |  |  |  | * | * |  |  | * |  |  | * |  |  |  | * |
